# Chest Radiograph Findings in Childhood Pneumonia Cases From the Multisite PERCH Study

**DOI:** 10.1093/cid/cix089

**Published:** 2017-05-29

**Authors:** Nicholas Fancourt, Maria Deloria Knoll, Henry C. Baggett, W. Abdullah Brooks, Daniel R. Feikin, Laura L. Hammitt, Stephen R. C. Howie, Karen L. Kotloff, Orin S. Levine, Shabir A. Madhi, David R. Murdoch, J. Anthony G. Scott, Donald M. Thea, Juliet O. Awori, Breanna Barger-Kamate, James Chipeta, Andrea N. DeLuca, Mahamadou Diallo, Amanda J. Driscoll, Bernard E. Ebruke, Melissa M. Higdon, Yasmin Jahan, Ruth A. Karron, Nasreen Mahomed, David P. Moore, Kamrun Nahar, Sathapana Naorat, Micah Silaba Ominde, Daniel E. Park, Christine Prosperi, Somwe wa Somwe, Somsak Thamthitiwat, Syed M. A. Zaman, Scott L. Zeger, Katherine L. O’Brien, for the PERCH Study Group, Katherine L. O’Brien, Orin S. Levine, Maria Deloria Knoll, Daniel R. Feikin, Andrea N. DeLuca, Amanda J. Driscoll, Nicholas Fancourt, Wei Fu, Laura L. Hammitt, Melissa M. Higdon, E. Wangeci Kagucia, Ruth A. Karron, Mengying Li, Daniel E. Park, Christine Prosperi, Zhenke Wu, Scott L. Zeger, Nora L. Watson, Jane Crawley, David R. Murdoch, W. Abdullah Brooks, Hubert P. Endtz, Khalequ Zaman, Doli Goswami, Lokman Hossain, Yasmin Jahan, Hasan Ashraf, Stephen R. C. Howie, Bernard E. Ebruke, Martin Antonio, Jessica McLellan, Eunice Machuka, Arifin Shamsul, Syed M.A. Zaman, Grant Mackenzie, J. Anthony G. Scott, Juliet O. Awori, Susan C. Morpeth, Alice Kamau, Sidi Kazungu, Micah Silaba Ominde, Karen L. Kotloff, Milagritos D. Tapia, Samba O. Sow, Mamadou Sylla, Boubou Tamboura, Uma Onwuchekwa, Nana Kourouma, Aliou Toure, Shabir A. Madhi, David P. Moore, Peter V. Adrian, Vicky L. Baillie, Locadiah Kuwanda, Azwifarwi Mudau, Michelle J. Groome, Nasreen Mahomed, Henry C. Baggett, Somsak Thamthitiwat, Susan A. Maloney, Charatdao Bunthi, Julia Rhodes, Pongpun Sawatwong, Pasakorn Akarasewi, Donald M. Thea, Lawrence Mwananyanda, James Chipeta, Phil Seidenberg, James Mwansa, Somwe wa Somwe, Geoffrey Kwenda

**Affiliations:** 1 Department of International Health, International Vaccine Access Center, Johns Hopkins Bloomberg School of Public Health, Baltimore, Maryland;; 2 Murdoch Children’s Research Institute and; 3 Royal Children’s Hospital, Melbourne, Australia;; 4 Global Disease Detection Center, Thailand Ministry of Public Health–US Centers for Disease Control and Prevention Collaboration, Nonthaburi;; 5 Division of Global Health Protection, Center for Global Health, Centers for Disease Control and Prevention, Atlanta, Georgia;; 6 Department of International Health, Johns Hopkins Bloomberg School of Public Health, Baltimore, Maryland;; 7 International Centre for Diarrhoeal Disease Research, Bangladesh, Dhaka and Matlab;; 8 Division of Viral Diseases, National Center for Immunizations and Respiratory Diseases, Centers for Disease Control and Prevention, Atlanta, Georgia;; 9 Kenya Medical Research Institute–Wellcome Trust Research Programme, Kilifi;; 10 Medical Research Council Unit, Basse, The Gambia;; 11 Department of Paediatrics, University of Auckland and; 12 Centre for International Health, University of Otago, Dunedin, New Zealand;; 13 Division of Infectious Disease and Tropical Pediatrics, Department of Pediatrics, Center for Vaccine Development, Institute of Global Health, University of Maryland School of Medicine,Baltimore;; 14 Bill & Melinda Gates Foundation, Seattle, Washington;; 15 Medical Research Council, Respiratory and Meningeal Pathogens Research Unit and; 16 Department of Science and Technology/National Research Foundation, Vaccine Preventable Diseases Unit, University of the Witwatersrand, Johannesburg, South Africa;; 17 Department of Pathology, University Otago and; 18 Microbiology Unit, Canterbury Health Laboratories, Christchurch, New Zealand;; 19 Department of Infectious Disease Epidemiology, London School of Hygiene & Tropical Medicine, United Kingdom;; 20 Center for Global Health and Development, Boston University School of Public Health, Massachusetts;; 21 Department of Pediatrics, Division of Emergency Medicine, Johns Hopkins School of Medicine, Baltimore, Maryland;; 22 Spokane Emergency Physicians, Washington;; 23 Department of Paediatrics and Child Health, University of Zambia School of Medicine and; 24 University Teaching Hospital, Lusaka, Zambia;; 25 Department of Epidemiology, Johns Hopkins Bloomberg School of Public Health, Baltimore, Maryland;; 26 Centre pour le Développement des Vaccins (CVD-Mali), Bamako, Mali;; 27 Department of International Health, Center for Immunization Research, Johns Hopkins Bloomberg School of Public Health, Baltimore, Maryland;; 28 Department of Diagnostic Radiology and; 29 Department of Paediatrics & Child Health, Chris Hani Baragwanath Academic Hospital and University of the Witwatersrand, Johannesburg, South Africa;; 30 Milken Institute School of Public Health, Department of Epidemiology and Biostatistics, George Washington University, DC;; 31 London School of Hygiene & Tropical Medicine, United Kingdom; and; 32 Department of Biostatistics, Johns Hopkins Bloomberg School of Public Health, Baltimore, Maryland; 33 (Johns Hopkins Bloomberg School of Public Health, Baltimore, Maryland); 34 (Bill & Melinda Gates Foundation, Seattle, Washington); 35 (Centers for Disease Control and Prevention (CDC)Atlanta, Georgia); 36 (The Emmes Corporation, Rockville, Maryland); 37 (Nuffield Department of Clinical Medicine, University of Oxford, United Kingdom); 38 (University of Otago, Christchurch, New Zealand); 39 (ICDDR, b, Dhaka and Matlab, Bangladesh); 40 (Medical Research Council, Basse, The Gambia); 41 (KEMRI-Wellcome Trust Research Programme, Kilifi, Kenya); 42 (Division of Infectious Disease and Tropical Pediatrics, Department of Pediatrics, Center for Vaccine Development, Institute of Global Health, University of Maryland School of Medicine, Baltimore, Maryland and Centre pour le Développement des Vaccins (CVD-Mali), Bamako, Mali); 43 (Respiratory and Meningeal Pathogens Research Unit, University of the Witwatersrand, Johannesburg, South Africa); 44 (Thailand Ministry of Public Health–US CDC Collaboration, Nonthaburi, Thailand); 45 (Boston University School of Public Health, Boston, Massachusetts and University Teaching Hospital, Lusaka, Zambia

**Keywords:** chest radiograph, pneumonia, pediatrics, signs and symptoms, mortality, .

## Abstract

**Background.:**

Chest radiographs (CXRs) are frequently used to assess pneumonia cases. Variations in CXR appearances between epidemiological settings and their correlation with clinical signs are not well documented.

**Methods.:**

The Pneumonia Etiology Research for Child Health project enrolled 4232 cases of hospitalized World Health Organization (WHO)–defined severe and very severe pneumonia from 9 sites in 7 countries (Bangladesh, the Gambia, Kenya, Mali, South Africa, Thailand, and Zambia). At admission, each case underwent a standardized assessment of clinical signs and pneumonia risk factors by trained health personnel, and a CXR was taken that was interpreted using the standardized WHO methodology. CXRs were categorized as abnormal (consolidation and/or other infiltrate), normal, or uninterpretable.

**Results.:**

CXRs were interpretable in 3587 (85%) cases, of which 1935 (54%) were abnormal (site range, 35%–64%). Cases with abnormal CXRs were more likely than those with normal CXRs to have hypoxemia (45% vs 26%), crackles (69% vs 62%), tachypnea (85% vs 80%), or fever (20% vs 16%) and less likely to have wheeze (30% vs 38%; all *P* < .05). CXR consolidation was associated with a higher case fatality ratio at 30-day follow-up (13.5%) compared to other infiltrate (4.7%) or normal (4.9%) CXRs.

**Conclusions.:**

Clinically diagnosed pneumonia cases with abnormal CXRs were more likely to have signs typically associated with pneumonia. However, CXR-normal cases were common, and clinical signs considered indicative of pneumonia were present in substantial proportions of these cases. CXR-consolidation cases represent a group with an increased likelihood of death at 30 days post-discharge.

## Summary

In the Pneumonia Etiology Research for Child Health study, abnormal chest radiographs (CXRs) in cases were associated with hypoxemia, crackles, tachypnea, and fever. Overall, 54% of CXRs were abnormal (site range, 35%–64%). Consolidation on CXR was associated with an increased risk of mortality.

The diagnosis of pneumonia in children is challenging because there is no single method with high sensitivity and high specificity [1, 2]. Clinical assessments for diagnosing pneumonia are sensitive but nonspecific, resulting in the inclusion of many nonpneumonia cases [3–5]. The use of chest radiographs (CXRs) to identify pneumonia cases is also imperfect but understood to identify fewer false-positive cases, especially for *Haemophilus influenzae* type b (Hib) and *Streptococcus pneumoniae* disease [6, 7]. How accurately standardized CXR definitions correlate to clinical signs and risk factors for pneumonia is less well described, especially in differing geographic areas where the predominant pathogens can vary. We aimed to describe the CXR findings of clinically diagnosed pneumonia cases in the Pneumonia Etiology Research for Child Health (PERCH) study and determine if there were differences in findings by geography, epidemiological setting, particular clinical signs, or pneumonia risk factors.

## METHODS

### Data Collection

The PERCH study is a multicountry, standardized, case-control study of the causes and risk factors of childhood pneumonia. Nine study sites were located in 7 countries: Dhaka and Matlab, Bangladesh; Basse, the Gambia; Kilifi, Kenya; Bamako, Mali; Soweto, South Africa; Nakhon Phanom and Sa Kaeo, Thailand; and Lusaka, Zambia. These sites include the geographic regions where the vast majority of severe and fatal pneumonia cases occur and represent a diverse range of epidemiological contexts [8].

Cases were hospitalized children aged 1–59 months with World Health Organization (WHO)–defined severe or very severe pneumonia [5, 9]. Severe pneumonia was defined as having cough and/or difficulty in breathing and lower chest wall indrawing. Very severe pneumonia was defined as cough and/or difficulty in breathing and at least 1 of the following “danger signs”: central cyanosis, difficulty breastfeeding/drinking, vomiting everything, convulsions, lethargy, unconsciousness, or head nodding. Case exclusion criteria were hospitalization within the previous 14 days, having been discharged as a PERCH case within the past 30 days, not residing in the study catchment area, or resolution of lower chest wall indrawing following bronchodilator therapy for those with severe pneumonia and wheeze. Standardization of clinical procedures used for participant enrollment and collection of PERCH data was achieved and maintained through on-site training for health-worker personnel, the use of a training website and training materials, and intermittent evaluations and competency assessments throughout the study [10].

A CXR was obtained from each case as soon as possible after clinical evaluation and study enrollment. All sites underwent a prestudy assessment of radiography facilities and procedures to review quality and safety and to standardize the collection of CXRs. Most sites used digital CXR imaging equipment, except Zambia and Matlab where analog techniques were used; analog images were scanned into digital format and all files managed according to a standardized operating procedure. Standardized methods for the collection and interpretation of chest radiographs are detailed elsewhere [11]. Each CXR was randomly assigned to be assessed by 2 members of a reading panel, comprised of 14 radiologists and pediatricians from study sites trained in the WHO methodology for the standardized interpretation of pediatric CXRs [7]. Two randomly selected members of a 4-person arbitration panel, consisting of experienced radiologists, resolved discordant interpretations by further blinded interpretations and a final consensus discussion if needed. Readers and arbitrators were unaware of the clinical and demographic information associated with each CXR, and readers did not interpret CXRs from their own site. Conclusions for CXRs were (1) consolidation (ie, alveolar consolidation, including pleural effusion if present) only; (2) other infiltrate only; (3) both consolidation and other infiltrate; (4) normal (no consolidation or infiltrate); and (5) uninterpretable for consolidation and/or other infiltrate. Additional conclusions derived from these 5 categories were (a) abnormal (1, 2, or 3), (b) any consolidation (1 or 3), and (c) any other infiltrate (2 or 3). For cases with multiple CXRs, the conclusion of the first interpretable CXR was used. CXR conclusions were excluded if the CXR was taken more than 72 hours after admission to avoid bias from possible nosocomial complications.

### Analyses

Results from the 2 Thailand sites were combined because they had similar epidemiologic and demographic characteristics. Results from sites with a high prevalence of human immunodeficiency virus (HIV) infection (South Africa and Zambia) were stratified by HIV status where relevant. HIV status was assumed to be negative for 385 cases where HIV status was unknown (HIV testing was not performed and/or there was no record of the child’s HIV history or maternal HIV history). Age was categorized as 1–5 months, 6–11 months, 12–23 months, and 24–59 months. Vaccination status was defined based on the number of doses a child received and the age at first dose or age at vaccine introduction in the community. Complete vaccination for pneumococcal conjugate vaccine was defined as 3 or more doses, or as 2 doses if there was at least 8 weeks between doses and the child was aged <9 months at enrollment or >12 months at the time of the first dose, or 1 or more doses if the age at any of the doses, or age at introduction, was ≥24 months. Complete vaccination for Hib conjugate vaccine was defined as 3 or more doses, or 1 or more doses for a child aged >12 months at first dose. Antibiotic pretreatment was defined by having either a positive serum bioassay or documented administration of antibiotics on the day of admission at the referral or study hospital prior to blood culture collection. Severe malnutrition was defined as a weight-for-age *z* score less than −3 below the median of the WHO child growth standards (WHO Anthro, version 3.2.2, January 2011). Moderate malnutrition was defined as a *z* score between −3 and −2 below the median of the WHO child growth standards. Tachypnea was defined as a respiratory rate ≥60 breaths per minute for children aged <2 months, ≥50 for children 2–11 months, and ≥40 for children 12–59 months. Tachycardia was defined as >160 beats per minute (bpm) for children aged 1–11 months, >150 bpm for children 12–35 months, and >140 bpm for children 36–59 months. Hypoxemia at admission was defined as either a room air pulse oximetry reading of <90% at the 2 sites at elevation (Zambia and South Africa) or <92% at all other sites or as a child treated with supplemental oxygen on admission but without a room air pulse oximetry reading. Nonpneumonia admission diagnoses were recorded.

Analyses were restricted to cases with interpretable CXRs. Distributions of categorical variables were compared using the Pearson χ^2^ statistic. Logistic regression was used to estimate odds ratios of CXR outcomes for predictors of clinical signs and risk factors, first in univariate models stratified by site to evaluate heterogeneity, and then overall adjusted for site, age, pneumonia severity, and HIV status. To assess the independent effects of variables potentially correlated with each other, such as findings on clinical examination (eg, tachypnea and hypoxemia) or nonpneumonia admission diagnoses (eg, bronchiolitis and asthma), multivariable logistic regression models were examined including all variables for danger signs and clinical signs (for very severe cases), clinical signs, and admission diagnoses. Analyses were completed using Stata 12.1 (Stata Corporation, College Station, Texas).

### Ethical Considerations

The institutional review board or ethical review committee at each study site institution and at the Johns Hopkins Bloomberg School of Public Health approved the PERCH study protocol. Parents or guardians of all participants provided written informed consent.

## RESULTS

**Figure 1.**
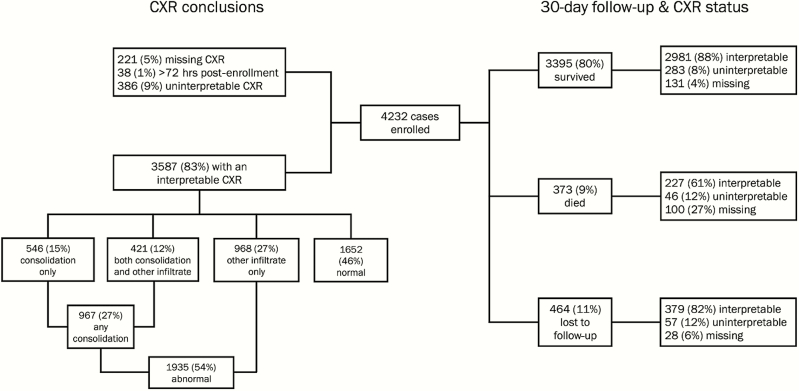
Flow diagram of case enrollment, chest radiograph (CXR) conclusions, and CXR status stratified by 30-day follow-up findings.

**Figure 2.**
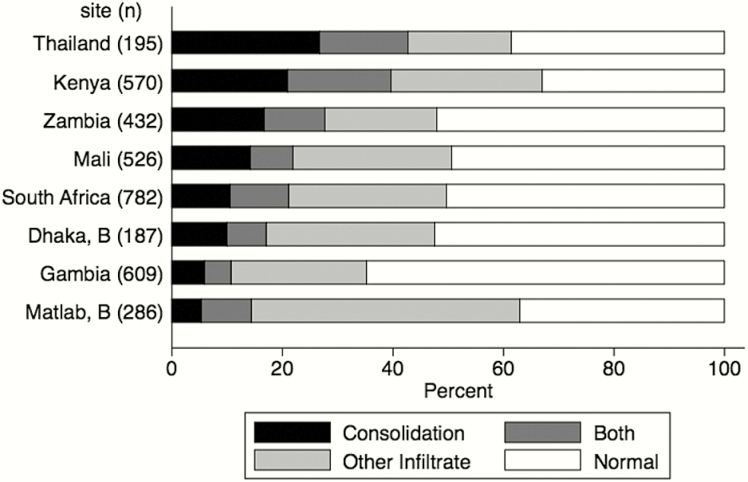
Distribution of chest radiograph conclusions by site, for interpretable images only (n = 3587). Abbreviation: B, Bangladesh; Both, both consolidation and other infiltrate.

A total of 3973/4232 (94%) cases had a CXR conclusion and of these 386 (10%) were uninterpretable (range, 4% in Gambia and Thailand to 20% in Zambia), leaving 3587 cases for analyses (Figure 1). Overall, 54% of interpretable CXRs were abnormal (site range from 35% in Matlab to 64% in HIV-negative cases from South Africa; Figure 2 and Table 1). Of these, 50% had consolidation (either alone or with other infiltrate) and 50% had other infiltrate only. Those with abnormal CXRs were more likely to be HIV infected (19% vs 5%) and aged <24 months (88% vs 83%; Table 1). However, the relationship between age and CXR outcome differed by severity strata. Among severe pneumonia cases, the youngest children (aged 1–5 months) were less likely to have an abnormal CXR compared to those in older age categories, and among very severe pneumonia cases, the youngest children were more likely to have an abnormal CXR (Supplemental Table 1).

**Table 1.** Distribution of Signs and Pneumonia Risk Factors by Chest Radiograph Findings Among Children Aged 1–59 Months Hospitalized With World Health Organization Defined Severe or Very Severe Pneumonia

		**All CXRs**			**Abnormal CXRs**		
	N	Normal	Abnormal	*P* Value^a^	Any Consolidation	Other Infiltrate Only	*P* Value^a^
Total (row %)	3587	1652 (46.1)	1935 (53.9)	--	967 (50.0)	968 (50.0)	--
Duration of illness (median days [interquartile range])	3568	3 (2–4)	3 (2–5)	<.001	3 (2–6)	3 (2–5)	<.001
Study site (row %)				<.001			<.001
Gambia	609	319 (52.4)	290 (47.6)		104 (35.9)	186 (64.1)	
Mali	526	273 (51.9)	253 (48.1)		146 (57.7)	107 (42.3)	
Zambia HIV−	366	158 (43.2)	208 (56.8)		135 (64.9)	73 (35.1)	
Zambia HIV+	66	8 (12.1)	58 (87.9)		50 (86.2)	8 (13.8)	
South Africa HIV−	681	246 (36.1)	435 (63.9)		250 (57.5)	185 (42.5)	
South Africa HIV+	101	12 (11.9)	89 (88.1)		60 (67.4)	29 (32.6)	
Kenya	570	286 (50.2)	284 (49.8)		121 (42.6)	163 (57.4)	
Thailand	195	96 (49.2)	99 (50.8)		43 (43.4)	56 (56.6)	
Bangladesh, Matlab	286	185 (64.7)	101 (35.3)		31 (30.7)	70 (69.3)	
Bangladesh, Dhaka	187	69 (36.9)	118 (63.1)		27 (22.9)	91 (77.1)	
Clinical and risk factor findings (column %)							
Age category, mo				.001			<.001
1–5	1414	653 (39.5)	761 (39.3)		417 (43.1)	344 (35.5)	
6–11	818	350 (21.2)	468 (24.2)		236 (24.4)	232 (24.0)	
12–23	842	375 (22.7)	467 (24.1)		197 (20.4)	270 (27.9)	
24–59	513	274 (16.6)	239 (12.4)		117 (12.1)	122 (12.6)	
Pneumonia severity				.10			<.001
Severe	2483	1121 (67.9)	1362 (70.4)		634 (65.6)	728 (75.2)	
Very severe	1104	531 (32.1)	573 (29.6)		333 (34.4)	240 (24.8)	
Danger signs^b^							
Head nodding (n = 1103)	570	235 (44.3)	335 (58.5)	<.001	209 (62.8)	126 (52.5)	.014
Central cyanosis (n = 1103)	71	19 (3.6)	52 (9.1)	<.001	27 (8.1)	25 (10.4)	.34
Convulsions (n = 1103)	203	145 (27.3)	58 (10.1)	<.001	28 (8.4)	30 (12.5)	.11
Lethargy (n = 1104)	357	183 (34.5)	174 (30.4)	.15	108 (32.4)	66 (27.5)	.21
Difficulty feeding (n = 1101)	243	110 (20.8)	133 (23.3)	.33	84 (25.3)	49 (20.4)	.17
Vomiting (n = 1102)	104	68 (12.9)	36 (6.3)	<.001	14 (4.2)	22 (9.2)	.016
Other clinical signs and history							
HIV positive (n = 1214)^c^	167	20 (4.7)	147 (18.6)	<.001	110 (22.2)	37 (12.5)	<.001
Hypoxemia (n = 3577)^d^	1287	425 (25.8)	862 (44.6)	<.001	500 (51.8)	362 (37.4)	<.001
Tachypnea (n = 3557)^e^	2938	1309 (79.7)	1629 (85.1)	<.001	827 (86.6)	802 (83.6)	.07
Crackles (n = 3569)	2353	1027 (62.3)	1326 (69.0)	<.001	647 (67.4)	679 (70.7)	.12
Wheeze (n = 3564)	1206	631 (38.3)	575 (30.0)	<.001	207 (21.6)	368 (38.4)	<.001
Grunting (n = 3568)	598	271 (16.5)	327 (17.0)	.67	203 (21.1)	124 (12.9)	<.001
Nasal flaring (n = 3581)	2094	887 (53.7)	1207 (62.5)	<.001	680 (70.5)	527 (54.6)	<.001
Tachycardia (n = 3579)^f^	1830	818 (49.6)	1012 (52.4)	.09	542 (56.2)	470 (48.7)	<.001
Temperature ≥38.5°C	637	257 (15.6)	380 (19.6)	.001	219 (22.6)	161 (16.6)	<.001
Weight-for-age (n = 3574)^g^				<.001			<.001
Normal	2427	1224 (74.3)	1203 (62.5)		569 (59.1)	634 (65.8)	
Moderate	635	261 (15.8)	374 (19.4)		186 (19.3)	188 (19.5)	
Severe	512	163 (9.9)	349 (18.1)		208 (21.6)	141 (14.6)	
Vaccine status (n = 1908)^h^				.91			.20
None	250	120 (13.3)	130 (12.9)		68 (15.0)	62 (11.3)	
Complete Hib, no PCV	618	296 (32.7)	322 (32.1)		139 (30.5)	183 (33.3)	
Complete PCV	1040	488 (54.0)	552 (55.0)		248 (54.5)	304 (55.4)	
Antibiotic pretreatment (n = 3370)^i^	1356	553 (35.9)	803 (43.9)	<.001	456 (49.5)	347 (38.3)	<.001
Died within 30 days (n = 3208)	227	73 (4.9)	154 (9.0)	<.001	113 (13.5)	41 (4.7)	<.001

Restricted to cases with an interpretable chest radiograph finding (n = 3587); for variables missing observations, a total n for that variable is shown in parentheses.

Abbreviations: CXR, chest radiograph; Hib, *Haemophilus influenzae* type b conjugate vaccine; HIV, human immunodeficiency virus; PCV, Pneumococcal conjugate vaccine.

^a^Pearson χ^2^ test for all categorical variables. Two-sample *t* test with equal variances for comparison of means of duration of illness.

^b^Very severe cases only.

^c^South Africa and Zambia sites only.

^d^Hypoxemia = room air pulse oximetry reading of <90% if at elevation (Zambia and South Africa) or <92% at all other sites. If a room air oxygen saturation reading was not available and the child was on supplemental oxygen, they were considered hypoxemic.

^e^Tachypnea = respiratory rate ≥60 breaths per minute if age <2 months, ≥50 if 2–11 months, and ≥40 if 12–59 months.

^f^Tachycardia = heart rate >160 beats per minute (bpm) if age 1–11 months, >150 bpm if 12–35 months, and >140 bpm if 36–59 months.

^g^Weight-for-age = severe if less than −3 *z* scores, moderate if between −3 and −2 *z* scores below WHO median.

^h^Vaccine comparison excludes those children too young (<4 months) to have completed a full biologic course of Hib and/or PCV vaccines, as these children by definition are considered to be “unvaccinated” but are not an appropriate comparison group for those who have been vaccinated. Complete vaccination for PCV was defined as 3 or more doses, or 2 doses if there was at least 8 weeks between doses and the child was aged <9 months at enrollment or >12 months at the time of the first dose, or 1 or more doses if the age at any of the doses, or age at introduction, was ≥24 months. Complete vaccination for Hib conjugate vaccine was defined as 3 or more doses, or 1 or more doses for a child aged >12 months at first dose.

^i^Antibiotic pretreatment was defined by having either a positive serum bioassay or documented administration of antibiotics on the day of admission at the referral or study hospital prior to blood culture collection.

We evaluated differences between cases with abnormal and normal CXRs to assess the likelihood that those with normal CXRs truly had pneumonia. Cases with abnormal CXRs were significantly more likely to have central cyanosis, tachypnea, hypoxemia, crackles, nasal flaring, or temperature ≥38.5°C and were less likely to have vomiting, convulsions, or wheeze than cases with normal CXRs (all *P* ≤ .001; Table 1). Results were similar after controlling for site, age, pneumonia severity, and HIV (Supplemental Table 2). Cases with moderate and severe malnutrition, as measured by weight-for-age, had higher odds of an abnormal CXR compared to those without malnutrition (Supplemental Table 2). Despite these statistically significant differences, high proportions of the 1652 CXR-normal cases had clinical signs generally considered more indicative of pneumonia (Table 1), including crackles (62%), nasal flaring (54%), hypoxemia (26%), grunting (17%), and head nodding (14%). CXR-normal cases had a mean of 1.7 of these signs, with 858/1652 (52%) cases having 2 or more signs, including 266/1652 (16%) cases who had crackles and hypoxemia. These signs were absent in 243/1652 (15%) CXR-normal cases compared to 129/1935 (7%) abnormal CXR cases. Results were similar when the 631 cases with wheezing were excluded (Supplemental Table 3).

We investigated the independent association of clinical signs and admission diagnoses with CXR findings using logistic regression models adjusted for multiple covariates. The following 3 groups of variables likely to show collinearity were identified: clinical signs among all cases, clinical and danger signs among very severe cases, and admission diagnoses. To assess independent effects of variables within each group, we included all variables for each group in a regression model and adjusted for site, age, severity, and HIV status (Table 2). For clinical signs among all cases, tachypnea, hypoxemia, crackles, and fever were all independently associated with an abnormal CXR, while wheeze was associated with a normal CXR (all *P* < .01). Among admission diagnoses, meningitis, bronchiolitis, gastroenteritis, and asthma were independently associated with a normal CXR; developmental delay was associated with an abnormal CXR; and the odds of having consolidation compared to a normal CXR were lower if admitted with a diagnosis of bronchiolitis, gastroenteritis, or meningitis (all *P* < .05). Among very severe cases, the only danger sign independently significantly associated with a CXR finding was convulsions, which showed lower odds of an abnormal CXR (odds ratio, 0.49; 95% confidence interval, 0.31–0.77). However, 58/203 (29%) cases with convulsions and 1/10 (10%) cases with convulsions alone did have an abnormal CXR. Respiratory signs (hypoxemia, nasal flaring, tachypnea, and crackles) were all associated with an abnormal CXR among very severe cases (all *P* < .01).

**Table 2.** Independent Associations Between Chest Radiograph Findings and Clinical Signs, Risk Factors, and Admission Diagnoses Among Children 1–59 Months of age Hospitalized with WHO Severe and Very Severe Pneumonia

	Abnormal vs Normal (ref)		Any consolidation vs Other infiltrate only (ref)		Any consolidation vs Normal (ref)		Other infiltrate only vs Normal (ref)	
	Adjusted OR^a^	*P* Value	Adjusted OR^a^	*P* Value	Adjusted OR^a^	*P* Value	Adjusted OR^a^	*P* Value
**All cases**								
*Clinical signs*								
Hypoxemia^b^	1.94	<.001	1.13	.30	2.05	<.001	1.84	<.001
Nasal flaring	1.10	0.24	1.29	.03	1.29	.02	0.97	.74
Tachypnea^c^	1.40	.001	1.39	.02	1.67	<.001	1.20	.12
Crackles	1.41	<.001	1.01	.90	1.38	.001	1.39	.001
Wheeze	0.69	<.001	0.61	<.001	0.52	<.001	0.87	.18
Temperature ≥38.5℃	1.41	<.001	1.24	.10	1.60	<.001	1.28	.04
Grunting	1.07	0.55	1.25	.15	1.14	.34	0.98	.88
Tachycardia^d^	1.00	0.99	1.09	.40	1.02	.85	0.96	.64
*Admission diagnoses*								
Paraffin ingestion	1.34	.69	--		2.62	.20	--	
Meningitis^e^	0.30	<.001	0.67	.21	0.25	<.001	0.39	<.001
Sepsis	1.09	.72	1.03	.93	1.12	.70	1.08	.79
Shock^f^	1.10	.32	1.29	.04	1.29	.03	0.96	.71
Bronchiolitis	0.67	<.001	0.72	.04	0.59	<.001	0.78	.07
Asthma	0.71	.04	0.82	.45	0.63	.05	0.76	.15
Gastroenteritis	0.59	.003	0.99	.96	0.61	.02	0.59	.02
Developmental delay	4.32	.001	0.86	.71	4.49	.002	4.79	.001
**Very severe cases**								
*Clinical and danger signs*								
Head nodding	0.75	.17	1.68	.06	0.93	.75	0.55	.03
Central cyanosis	1.55	.16	0.85	.65	1.34	.43	1.67	.18
Inability to feed/drink	1.00	1.00	1.67	.03	1.25	.27	0.74	.20
Vomiting	0.66	.12	0.63	.25	0.47	.04	0.79	.47
Lethargy or unconsciousness	0.98	.93	1.69	.04	1.25	.28	0.76	.21
Convulsions	0.49	.002	0.86	.67	0.46	.007	0.51	.03
Hypoxemia^b^	1.63	.002	1.34	.16	1.79	.002	1.38	.10
Nasal flaring	1.61	.006	0.98	.95	1.68	.02	1.58	.04
Tachypnea^c^	1.59	.009	1.45	.17	2.04	.002	1.35	.17
Crackles	1.95	<.001	1.26	.26	1.96	<.001	1.85	.002
Wheeze	0.67	.03	0.63	.06	0.56	.008	0.87	.56
Temperature >38.5℃	1.24	.23	0.98	.93	1.30	.22	1.26	.30
Grunting	0.82	.31	1.01	.97	0.82	.40	0.84	.47
Tachycardia^d^	1.33	.05	1.38	.10	1.46	.03	1.15	.42

Abbreviations: OR, Odds ratio; WHO, World Health Organization.

^a^Within each of the three models (clinical signs; admission diagnoses; and danger signs and clinical signs among cases with very severe pneumonia), all indicated variables were included, regardless of univariate significance. The three models were developed to examine clinical characteristics with likely collinearity. Models are adjusted for age (in months), site (not stratified for HIV status), severity (‘All Cases’ models only), and HIV status; ‘no’ is the reference category for all variables.

^b^Hypoxemia=room air pulse-oximetry reading of <90% if at elevation (Zambia and South Africa) or <92% at all other sites. If a room air oxygen saturation reading was not available and the child was on supplemental oxygen they were also considered hypoxemic.

^c^Tachypnea=respiratory rate ≥60 breaths per minute if age <2 months, ≥50 if 2-11 months, and ≥40 if 12-59 months.

^d^Tachycardia=heart rate >160 beats per minute (bpm) if age 1-11 months, >150 bpm if 12-35 months, and >140 bpm if 36-59 months.

^e^Meningitis defined as an admission diagnosis of meningitis or a bulging fontanelle if age <18 months.

^f^Shock defined as capillary refill time of >3sec, or cool peripheries, or weak pulse with tachycardia.

We assessed the relationship between CXR findings and mortality (Figure 1 and Table 3). Those who died and had an interpretable CXR were twice as likely to have findings of consolidation (50% vs 24%) and less likely to have other infiltrates only (18% vs 28%) compared to those who survived. Of 3208 cases with an interpretable CXR and documented 30-day survival status, 837 (26%) had a CXR finding of any consolidation of which 113 died (case fatality ratio [CFR], 13.5%) compared to 874 cases with other infiltrate only among whom there were 41 deaths (CFR, 4.7%) and 1497 cases that were CXR-normal among whom there were 73 deaths (CFR, 4.9%; Table 3). These trends were similar when stratified by age (<12 months and ≥12 months; data not shown).

**Table 3.** Case Fatality Ratios by Chest Radiograph Findings for Cases with Known Survival Status at 30 Days Post Discharge^a^

		**Total Cases (N) and Case Fatality Ratio (%**)																	
**Chest radiograph finding**	**Total Deaths**	All sites		Gambia		Mali		Zambia		South Africa		Kenya		Thailand		Matlab		Dhaka	
		N	%	N	%	N	%	N	%	N	%	N	%	N	%	N	%	N	%
**Overall** ^a^																			
Interpretable	227	3208	7.1	580	4.5	510	12.4	246	24.0	661	5.6	555	5.4	190	4.7	282	0.4	184	1.1
Abnormal	154	1711	9.0	279	5.7	245	15.1	159	26.4	436	7.8	278	6.5	98	5.1	100	1.0	116	0.9
Normal	73	1497	4.9	301	3.3	265	9.8	87	19.5	225	1.3	277	4.3	92	4.4	182	0.0	68	1.5
Any consolidation	113	837	13.5	102	8.8	142	19.7	117	29.1	258	9.3	119	10.9	42	9.5	31	0.0	26	3.9
Other infiltrate only	41	874	4.7	177	4.0	103	8.7	42	19.1	178	5.6	159	3.1	56	1.8	69	1.4	90	0.0
Uninterpretable	46	329	14.0	27	0.0	67	19.4	64	34.4	89	9.0	26	7.7	9	0.0	38	2.6	9	0.0
Missing	100	231	43.3	2	50.0	77	57.1	59	74.6	35	11.4	35	17.1	20	0.0	1	0.0	2	50.0
**HIV negative** ^b^																			
Abnormal	118	1582	7.5		--		--	125	20.0	359	5.3		--		--		--		--
Normal	66	1474	4.5		--		--	81	17.3	214	0.0		--		--		--		--
Any consolidation	85	739	11.5		--		--	87	21.8	205	6.8		--		--		--		--
Other infiltrate only	33	843	3.9		--		--	38	15.8	154	3.3		--		--		--		--
**HIV positive** ^b^																			
Abnormal	36	129	27.9		--		--	34	50.0	77	19.5		--		--		--		--
Normal	7	23	30.4		--		--	6	50.0	11	27.3		--		--		--		--
Any consolidation	28	98	28.6		--		--	30	50.0	53	18.9		--		--		--		--
Other infiltrate only	8	31	25.8		--		--	4	50.0	24	20.8		--		--		--		--

Abbreviation: HIV, human immunodeficiency virus.

^a^Cases with unknown 30-day survival (n=464) are omitted from this analysis.

^b^Site-specific HIV analyses are restricted to sites with high prevalence only (South Africa and Zambia).

## DISCUSSION

The PERCH study allows for a comprehensive assessment of the association between clinical characteristics and CXR findings in childhood pneumonia cases under highly standardized clinical and radiographic interpretation conditions. We found that at the time of admission, those with WHO-defined severe or very severe pneumonia who had tachypnea, hypoxemia, crackles, or fever were more likely to have an abnormal CXR than those who did not have these findings, irrespective of other clinical signs. In contrast, those with convulsions or wheeze were less likely than those without these findings to have an abnormal CXR. These findings provide evidence that PERCH analyses restricted to cases with abnormal CXRs largely represent true pneumonia cases. CXR-normal cases likely include some children who have true pneumonia, as suggested by the 52% of these cases who had 2 or more findings of hypoxemia, crackles, head nodding, nasal flaring, or grunting; however, it is difficult to distinguish with certainty which of these cases have pneumonia and which do not. Thus, as expected, standardized CXR definitions for epidemiological studies have limited predictive value for clinical care because a substantial proportion of children with a normal CXR finding also have clinical signs or risk factors indicative of pneumonia.

The categorization of CXRs in this study followed an established methodology developed by the WHO for use in bacterial vaccine trials [7]. Previous evidence on correlations between clinical signs and radiological findings is mixed and comparisons are difficult because of varied definitions of “radiological pneumonia.” Some studies showed that tachypnea [12], hypoxemia [13], and crackles [14] on admission may be predictive of radiological pneumonia, while others found clinical signs to be poor predictors of radiographic findings [15, 16]. A recent metaanalysis indicated that no single clinical feature is sufficient to predict radiological pneumonia [17], although that analysis involved unstandardized CXR interpretation methods and CXR definitions. In contrast, our findings suggest that hypoxemia, tachypnea, crackles, and fever may predict the WHO standardized definition of CXR consolidation in a study where case enrollment used a standardized clinical definition known to have high sensitivity but low specificity (Table 2).

We identified substantial differences between sites in the distribution of CXR findings (Table 1 and Figure 2). Between 4% and 20% of CXRs from each site were uninterpretable, and although we used a more stringent definition of “uninterpretable for other infiltrate and/or consolidation” compared to the “uninterpretable for consolidation only” definition in most applications of the WHO methodology [18], high proportions of uninterpretable images highlight the challenges of achieving adequate radiographic images in resource-poor settings. The proportion of PERCH cases with a finding of consolidation (with or without other infiltrate) ranged, by site, from 11% to 37% in HIV-negative cases. Such differences are consistent with findings of consolidation in previous studies of pneumonia. In the Gambian pneumococcal conjugate vaccine trial, 17% of hospitalized pneumonia cases in the control arm had consolidation in the presence of Hib vaccine [19], while in the Bangladesh Hib vaccine case-control study, 26% of all hospitalized pneumonia cases, vaccinated and nonvaccinated combined, had consolidation [20]. The proportion of abnormal CXRs (consolidation or other infiltrate) among HIV-negative cases also varied by site from 35% to 64% (and thus a range of normal CXRs from 36% to 65%), while 88% of HIV-positive cases had an abnormal CXR in both South Africa and Zambia. The commonly held understanding is that etiology, severity, duration of infection, and host immune reaction are major predictors of CXR findings [21–23]. If this is true, observed differences in the distributions of CXR findings between sites likely reflect determinants of those CXR predictors, such as healthcare-seeking behaviors, availability of antibiotics, and environmental and living conditions. Epidemiologic studies that use CXR case definitions should recognize that differences between studies could reflect not only differences in pneumonia etiology but also variables that alter the timing of care seeking or host response to infection.

Age has an important interaction with CXR results and severity, as expected (Supplemental Table 1). Danger signs, which are not specific for respiratory disease, were more indicative of an abnormal CXR in younger children; we observed that among very severe cases, the youngest children (aged 1–5 months) were more likely to have an abnormal CXR than older children. However, more specific pneumonia findings (such as lower chest wall indrawing) are less associated with pneumonia in younger children because the physiologic compliance of the lower chest makes this finding more common in a variety of illnesses. Thus, among severe cases, we observed that an abnormal CXR was less likely among the youngest children compared to those in other age groups. These findings suggest that danger signs may not be useful criteria for diagnosing pneumonia in children aged >6 months, particularly in pneumonia studies where a low proportion of false positives is important. However, danger signs remain useful for bacterial treatment algorithms, since they will identify nonpneumonia cases that nevertheless require treatment.

We observed significant differences in case fatality ratios among HIV-negative cases depending on their CXR findings; those with consolidation on CXR were at significantly higher risk of death than those with a normal CXR or with other infiltrates only. The presence of other infiltrates did not alter the risk of death compared to a normal CXR appearance in children with severe or very severe pneumonia. This likely reflects the association between consolidation and bacterial pneumonia and the tendency for bacterial pneumonia to be more severe than viral pneumonia. An association between a CXR finding of “dense infiltrates” and mortality has also been documented in the Philippines [24]; in Fiji, CXR consolidation had a case fatality ratio of 6.8% using the WHO methodology [25]. Our findings are limited in that 100 cases who died were missing a CXR, the majority (92%) because death occurred before this could be obtained. If the distribution of CXR findings in these cases differed from the other cases who died, the difference in case fatality ratios may either be underestimated or overestimated. Nonetheless, these findings suggest that in order to further reduce the mortality burden of pneumonia, a focus on groups with a high prevalence of consolidation should be a priority.

Our findings have important methodological limitations. First, without a gold standard for pneumonia diagnosis, clinical signs and radiological findings may be correlated to each other without being correlated to true pneumonia. Second, if observers recording clinical signs were aware of a case’s CXR appearance, the independence of these measurements may be violated. Third, our definition of hypoxemia included those on supplemental oxygen, which may not reliably indicate true hypoxemia on admission. Finally, our study was not designed to assess the relationship between duration of illness and abnormal CXR findings, yet this is likely to have a strong influence on CXR positivity.

In summary, the WHO clinical pneumonia definitions were designed as a sensitive tool for the management of pneumonia cases. Our experience suggests that almost half of these cases do not have radiological evidence of pneumonia. Chest radiography continues to be a valuable method for case identification that is correlated to clinical signs of pneumonia and can be applied to a variety of pneumonia studies, including etiology studies. Yet, radiologic findings in cases of clinical pneumonia likely reflect a complex mix of etiology, healthcare-seeking patterns, antibiotic use, age, and underlying health conditions such as HIV and malnutrition.

## Supplementary Data

Supplementary materials are available at *Clinical Infectious Diseases* online. Consisting of data provided by the authors to benefit the reader, the posted materials are not copyedited and are the sole responsibility of the authors, so questions or comments should be addressed to the corresponding author.

## Supplementary Material

Supplementary_Data

## Notes

***Author contributions.*** N. F. performed the analysis, interpreted results, and drafted the manuscript. M. D. K. and K. L. O. assisted with analysis and interpretation. O. S. L., K. L. O., D. R. F., D. R. M., M. D. K., L. L. H., H. C. B., W. A. B., S. R. C. H., K. K. L., S. A. M., J. A. G. S., and D. M. T. conceived and designed the study and supervised study conduct. J. O. A., B. B. K., J. C., A. N. D., M. D., A. J. D., B. E. E., M. M. H., Y. J., N. M., D. P. M., K. N., S. N., D. E. P., C. P., M. S., S. W. S., S. T., and S. M. A. Z. were involved in study conduct. B. B. K., M. D., B. E. E., N. M., D. P. M., K. N., and M. S. O. were part of the PERCH Chest Radiograph Reading Panel. S. Z. provided statistical expertise and led the integrated etiology analysis. R. A. K. provided technical guidance. All authors reviewed and approved the manuscript. N. F. had full access to all the data in the study and had final responsibility for the decision to submit for publication.

***Acknowledgments.*** We offer sincere thanks to the patients and families who participated in this study. We are grateful to the following members of the original study teams for running the studies and collecting data:

***Financial support.*** PERCH was supported by a grant from the Bill & Melinda Gates Foundation to the International Vaccine Access Center, Department of International Health, Johns Hopkins Bloomberg School of Public Health (grant 48968). J. A. G. S. was supported by a clinical fellowship from the Wellcome Trust of Great Britain (098532). N. F. was supported by an International Fulbright Science and Technology Fellowship from the US Department of State.

***Supplement sponsorship.*** This article appears as part of the supplement “Pneumonia Etiology Research for Child Health (PERCH): Foundational Basis for the Primary Etiology Results,” sponsored by a grant from the Bill & Melinda Gates Foundation to the PERCH study of Johns Hopkins Bloomberg School of Public Health, Baltimore, Maryland.

***Potential conflicts of interest.*** M. D. K. has received funding for consultancies from Merck, Pfizer, and Novartis and grant funding from Merck. L. L. H. has received grant funding from Pfizer and GlaxoSmithKline. K. L. K. has received grant funding from Merck Sharp & Dohme. S. A. M. has received honorarium for advisory board membership from the Bill & Melinda Gates Foundation (BMGF), Pfizer, Medimmune, and Novartis; institutional grants from GSK, Novartis, Pfizer, Minervax and BMGF; and speakers bureau fees from Sanofi Pasteur and GSK. K. L. O. has received grant funding from GSK and Pfizer and participates on technical advisory boards for Merck, Sanofi Pasteur, PATH, Affinivax, and ClearPath. All other authors: No reported conflicts. The authors have submitted the ICMJE Form for Disclosure of Potential Conflicts of Interest. Conflicts that the editors consider relevant to the content of the manuscript have been disclosed.
