# Supplementary material for: Chest Radiograph Findings in Childhood Pneumonia Cases From the Multisite PERCH Study
Source: Clin Infect Dis. 2017 May 29;64(Suppl 3):S262–70. doi: 10.1093/cid/cix089 (PMC5447837; doi:10.1093/cid/cix089)
Supplement: Supplementary_Data [file cix089_suppl_Supplementary_Data.pdf]

Supplemental Table 1: Association of chest radiograph outcome (normal=ref vs. abnormal) with clinical signs, risk factors, and admission diagnoses overall and by site

Variable	Adjusted Odds Ratio [b]										
	Overall	Gambia	Mali	Zambia (HIV -)	Zambia (HIV +)	South Africa (HIV -)	South Africa (HIV +)	Kenya	Thailand	Matlab	Dhaka
Sex (ref=male)	1.16*	0.93*	0.96	0.80	--	1.66**	0.64	1.41*	1.54	1.07	1.29
Severity	0.82	0.92	0.52**	1.36	0.41	1.45*	0.33**	0.43**	1.88	1.29	1.37
Age category (months; ref=1-5)											
1-5 vs 6-11	1.19	1.27	1.10	1.19	1.04	1.20	2.70	1.24	0.62	1.29	1.38
1-5 vs 12-23	1.17	1.40	1.46	1.35	1.48	0.95	3.20	0.97	0.64	1.31	1.28
1-5 vs 24-59	0.84	0.93	0.91	0.64	1.04	0.61	1.10	0.65	0.71	1.94	0.59
Age category (months; ref=1-5), severe											
1-5 vs 6-11	1.36**	1.20	1.58	1.14	0.32	1.54	3.05	2.18*	0.47	1.39	1.42
1-5 vs 12-23	1.38**	1.37	2.09*	1.04	--	1.32	1.00	1.54	0.58	1.51	1.25
1-5 vs 24-59	1.14	1.29	1.69	0.67	0.35	0.68	1.00	1.11	0.72	2.03	0.50
Age category (months; ref=1-5), very severe											
1-5 vs 6-11	0.90	1.99	0.78	1.34	--	0.68	2.36	0.62	2.00	1.40	1.20
1-5 vs 12-23	0.88	1.49	0.94	2.29	0.90	0.48*	0.64	0.73	--	0.35	1.80
1-5 vs 24-59	0.42	0.29	0.37*	0.57	--	0.58	1.00	0.41*	0.67	2.80	1.80
**Danger signs**											
Head nodding	1.33*	2.78*	1.33	0.83	--	1.09	3.33	1.86*	0.33	--	0.42
Central cyanosis	2.57**	--	4.18**	1.26	0.40	4.62	0.22	0.50	4.40	--	--
Inability to feed/drink	1.28	0.65	1.27	1.12	0.10	1.62	0.72	2.39**	3.44	--	1.06
Vomiting	0.52**	0.44	2.35	--	--	0.56	--	0.43**	0.50	--	--
Lethargy/unconscious	0.99	0.84	0.54*	1.38	0.55	0.70	2.40	1.30	1.20	--	4.45
Convulsions	0.43**	0.16**	0.47*	0.76	0.04*	0.53	0.22	0.28**	0.47	--	2.00
**Clinical signs**											
Tachypnea [a]	1.51**	2.02**	1.12	1.74**	34.20**	1.03	0.98	2.37**	0.84	1.90	--

Variable	Adjusted Odds Ratio [b]										
	**Overall**	**Gambia**	**Mali**	**Zambia (HIV -)**	**Zambia (HIV +)**	**South Africa (HIV -)**	**South Africa (HIV +)**	**Kenya**	**Thailand**	**Matlab**	**Dhaka**
Hypoxemia [b]	2.08**	3.27**	2.28**	3.05**	2.86	1.12	0.31	2.71**	1.68	0.68	1.13
Crackles	1.47**	1.42**	2.24**	0.92	1.07	1.22	1.09	2.00**	1.20	1.73	1.72
Wheeze	0.70**	0.86	0.61*	0.82	1.00	0.55**	0.52	1.06	0.39**	3.35	1.03
Grunting	1.21**	1.40	1.06	0.95	1.00	1.00	0.66	2.01	2.50	3.16	--
Nasal Flaring	1.24**	1.35	1.63*	1.01	1.89	0.86	1.50	1.97**	0.50*	0.91	0.72
Tachycardia [c]	1.10	1.16	1.18	1.03	0.78	0.95	0.56	1.35**	1.02	0.74	2.15
Temperature	1.43**	2.00**	1.26	1.62*	1.83	1.14	--	1.19	1.71	0.51	0.87
Weight-for-age (ref=normal) [d]											
Moderate	1.42**	1.55*	1.09	1.43	1.02	1.74*	0.90	1.32	2.04	1.55	1.54
Severe	1.94**	1.38	3.04**	1.28	4.00	2.42**	1.50	1.59*	2.00	1.81	3.07*
Height-for-age (ref=normal) [d]											
Moderate	1.51**	1.05	2.55**	1.30	0.86	1.20	0.60	1.70*	1.23	2.20*	1.44
Severe	1.36**	0.80	3.07**	1.19	2.57	1.58*	1.17	1.03	3.51	1.32	1.14
Weight-for-height (ref=normal) [d]											
Moderate	1.28*	1.21	1.05	0.80	1.74	1.51	0.52	1.82*	2.67*	1.20	1.42
Severe	1.50**	1.30	2.04**	3.98**	1.11	1.31	1.10	1.27	1.26	1.07	3.87
**History**											
Died within 30 days	1.74**	1.77	1.64**	1.20	--	--	0.65	1.53	1.18	--	0.58
Antibiotic pretreatment [e]	1.15**	1.30	0.80	1.56	--	1.75**	0.52	0.90	--	0.79	2.12
Hib & PCV vaccination (ref=none) [f]											
Complete Hib, no PCV	1.18	1.79	0.84	--	--	1.72	1.11	0.15	--	--	2.13
Complete PCV	1.02	2.06	0.57	--	--	0.72	--	0.26	--	--	--

Abbreviations: Hib=*Haemophilus influenzae* type b conjugate vaccine; PCV=Pneumococcal conjugate vaccine; HIV=human immunodeficiency virus.

[a] Adjusted for age (in months), pneumonia severity, HIV status. Overall model is also adjusted for site (not stratified by HIV status).

[b] Tachypnea=respiratory rate ≥60 breaths per minute for children <2 months of age, ≥50 for children 2-11 months, and ≥40 for children 12-59 months.

[b] Hypoxemia=room air pulse-oximetry reading of <90% if at elevation (Zambia and South Africa) or <92% at all other sites. If a room air oxygen saturation reading was not available and the child was on supplemental oxygen they were also considered hypoxemic.

[c] Tachycardia=heart rate >160 beats per minute (bpm) if age 1-11 months, >150 bpm if age 12-35 months, and >140 bpm if age 36-59 months.

[d] Weight-for-age, height-for-age, and weight-for-height=severe if <-3 z-scores, moderate if between -3 and -2 z-scores below WHO median.

[e] Antibiotic pretreatment was defined by having either a positive serum bioassay or documented administration of antibiotics on the day of admission at the referral or study hospital prior to blood culture collection .
[f] Vaccine comparison excludes those children too young (<4 months) to have completed a full biologic course of Hib and/or PCV vaccines, as these children by definition are considered to be 'unvaccinated' but are not an appropriate comparison group for those who have been vaccinated. Complete vaccination for PCV was defined as 3 or more doses, or 2 doses if there was at least 8 weeks between doses and the child was <9 months of age at enrollment or >12 months of age at the time of the first dose, or 1 or more doses if the age at any of the doses, or age at introduction, was ≥24 months. Complete vaccination for Hib conjugate vaccine was defined as 3 or more doses, or 1 or more doses for a child older than 12 months at first dose.
* $p<0.05$, ** $p<0.01$; for binary (no/yes) variables, 'no' is the reference category.

Supplemental Table 2: Association of chest radiograph outcome with clinical signs, risk factors, and admission diagnoses among children 1-59 months of age hospitalized with WHO severe or very severe pneumonia

Case characteristic	Normal (ref) vs Abnormal		Other infiltrate only (ref) vs Any consolidation		Normal (ref) vs Any consolidation		Normal (ref) vs Other infiltrate only	
	OR	Adj OR [a]	OR	Adj OR	OR	Adj OR	OR	Adj OR
**Severe (ref) vs Very severe**	0.89	0.82*	1.59**	1.36**	1.11	0.97	0.70**	0.70**
**Danger signs** [b]								
Head nodding	1.77**	1.32	1.52*	1.50*	2.12**	1.54*	1.39*	1.05
Central cyanosis	2.68**	2.56**	0.76	0.68	2.37**	2.09*	3.13**	3.17**
Inability to feed/drink	1.15	1.26	1.32	1.56*	1.29	1.49*	0.98	0.99
Vomiting	0.45**	0.53**	0.43*	0.56	0.30**	0.39**	0.68	0.68
Lethargy or unconsciousness	0.83	1.01	1.23	1.42	0.90	1.14	0.73	0.85
Convulsions	0.31**	0.42**	0.62	0.75	0.25**	0.37**	0.40**	0.49**
**Clinical signs**								
Tachypnea [c]	1.46**	1.53**	1.26	1.46**	1.65**	1.85**	1.30*	1.26*
Hypoxemia [d]	2.31**	2.07**	1.80**	1.13	3.09**	2.21**	1.72**	1.93**
Crackles	1.35**	1.48**	0.86	1.05	1.25**	1.49**	1.46**	1.41**
Wheeze	0.69**	0.71**	0.44**	0.61**	0.44**	0.54**	1.00	0.88
Grunting	1.04	1.21	1.80**	1.35*	1.35**	1.36*	0.75*	1.05
Nasal flaring	1.44**	1.24**	1.99**	1.37**	2.06**	1.46**	1.03	1.06
Tachycardia [e]	1.12	1.10	1.36**	1.15	1.30**	1.17	0.96	1.02
Temperature ≥38.5℃	1.33**	1.46**	1.47**	1.41**	1.59**	1.75**	1.08	1.25
**Risk factors**								
HIV positive [f]	5.87**	4.52**	3.48**	2.24**	9.37**	6.00**	2.70**	2.54**
Antibiotic pretreatment [g]	1.40**	1.15	1.58**	0.99	1.75**	1.20	1.11	1.14
Weight-for-age (ref=normal) [h]								
Moderate	1.46**	1.46**	1.10	1.21	1.53**	1.56**	1.39**	1.35**
Severe	2.18**	1.98**	1.64**	1.45**	2.75**	2.42**	1.67**	1.63**
PCV & Hib Vaccine (ref=none) [i]								
Complete Hib, no PCV	1.00	1.24	0.69	0.73	0.83	0.96	1.20	1.59

Case characteristic	Normal (ref) vs Abnormal		Other infiltrate only (ref) vs Any consolidation		Normal (ref) vs Any consolidation		Normal (ref) vs Other infiltrate only	
	OR	Adj OR [a]	OR	Adj OR	OR	Adj OR	OR	Adj OR
Complete PCV	1.04	1.11	0.74	0.59	0.90	0.78	1.21	1.61
**Admission diagnoses**								
Paraffin ingestion	1.43	1.50	--	--	2.87	2.93	--	--
Meningitis [j]	0.32**	0.34**	0.96	0.64	0.31**	0.28**	0.33**	0.43**
Sepsis	1.02	1.05	1.39	1.15	1.19	1.13	0.85	0.98
Shock [k]	1.07	1.12	1.62**	1.33*	1.34**	1.32*	0.83	0.96
Bronchiolitis	0.79*	0.70**	0.86	0.69*	0.73*	0.61**	0.85	0.83
Asthma	0.59**	0.72*	0.54*	0.81	0.42**	0.63*	0.77	0.77
Gastroenteritis	0.73	0.67*	1.33	1.03	0.84	0.69	0.63*	0.66
Developmental delay	3.70**	4.32**	1.00	0.97	3.70**	4.71**	3.70**	4.56**
Malaria with parasitemia >5000/uL [l]	0.22**	0.29**	1.93	1.71	0.29**	0.37*	0.15**	0.19**

Abbreviations: Hib=*Haemophilus influenzae* type b conjugate vaccine; PCV=Pneumococcal conjugate vaccine; OR=odds ratio; HIV=human immunodeficiency virus, WHO, World Health Organization.

[a] Adjusted for age (in months), site (not stratified for HIV status), HIV status, and pneumonia severity (except danger signs and severity models).

[b] Danger sign analyses were restricted to very severe cases only.

[c] Tachypnea=respiratory rate ≥60 breaths per minute for children <2 months of age, ≥50 for children 2-11 months, and ≥40 for children 12-59 months.

[d] Hypoxemia=room air pulse-oximetry reading of <90% if at elevation (Zambia and South Africa) or <92% at all other sites. If a room air oxygen saturation reading was not available and the child was on supplemental oxygen they were also considered hypoxemic.

[e] Tachycardia=heart rate >160 beats per minute (bpm) if age 1-11 months, >150 bpm if age 12-35 months, and >140 bpm if age 36-59 months.

[f] South Africa and Zambia sites only.

[g] Antibiotic pretreatment was defined by having either a positive serum bioassay or documented administration of antibiotics on the day of admission at the referral or study hospital prior to blood culture collection.

[h] Weight-for-age=severe if <-3 z-scores, moderate if between -3 and -2 z-scores below WHO median.

[i] Adjusted models for vaccine use do not include site, as there is little variation in vaccine use within sites making site collinear to vaccine use, and exclude those children too young (<4 months) to have completed a full biologic course of Hib and/or PCV vaccines, as these children by definition are considered to be ‘unvaccinated’ but are not an appropriate comparison group for those who have been vaccinated. Complete vaccination for PCV was defined as 3 or more doses, or 2 doses if there was at least 8 weeks between doses and the child was <9 months of age at enrollment or >12 months of age at the time of the first dose, or 1 or more doses if the age at any of the doses, or age at introduction, was ≥24 months. Complete

vaccination for Hib conjugate vaccine was defined as 3 or more doses, or 1 or more doses for a child older than 12 months at first dose.
[j] Meningitis defined as an admission diagnosis of meningitis or a bulging fontanelle if age <18 months.
[k] Shock defined as capillary refill time of >3sec, or cool peripheries, or weak pulse with tachycardia.
[l] Malaria analyses restricted to sites with endemic disease (Gambia, Mali, Kenya, and Zambia).
* p<0.05, ** p<0.01; for binary (no/yes) variables, ‘no’ is the reference category.

Supplemental Table 3: Distribution of signs and pneumonia risk factors by chest radiograph (CXR) findings among children 1-59 months of age hospitalized with WHO severe or very severe pneumonia, excluding cases with wheeze [a]

		All CXRs			Abnormal CXRs		
	N	Normal	Abnormal	p-value [b]	Any consolidation	Other infiltrate only	p-value [b]
Total (row %)	2358	1016 (43.1)	1342 (56.9)	--	752 (56.0)	590 (44.0)	--
**Clinical and risk factor findings (column %)**							
Age category				<.001			.16
1-5 months	1006	434 (42.7)	572 (42.6)		334 (44.4)	238 (40.3)	
6-11 months	533	202 (19.9)	331 (24.7)		188 (25.0)	143 (24.2)	
12-23 months	503	213 (21.0)	290 (21.6)		146 (19.4)	144 (24.4)	
24-59 months	316	167 (16.4)	149 (11.1)		84 (11.2)	65 (11.0)	
Pneumonia severity				<.001			.02
Severe	1520	607 (59.7)	913 (68.0)		491 (65.3)	422 (71.5)	
Very severe	838	409 (40.3)	429 (32.0)		261 (34.7)	168 (28.5)	
HIV positive [c]	168	23 (2.3)	145 (10.8)	<.001	114 (15.2)	31 (5.3)	<.001
Head nodding	350	128 (12.6)	222 (16.5)	.008	154 (20.5)	68 (11.5)	<.001
Central cyanosis	65	18 (1.8)	47 (3.5)	.01	26 (3.5)	21 (3.6)	.92
Hypoxemia [d]	919	277 (27.3)	642 (47.9)	<.001	400 (53.3)	242 (41.0)	<.001
Convulsions	227	161 (15.8)	66 (4.9)	<.001	32 (4.3)	34 (5.8)	.20
Lethargy	321	172 (16.9)	149 (11.1)	<.001	93 (12.4)	56 (9.5)	.10
Difficulty feeding	193	85 (8.4)	108 (8.1)	.78	70 (9.3)	38 (6.4)	.05
Vomiting	93	62 (6.1)	31 (2.3)	<.001	13 (1.7)	18 (3.1)	.11
Tachypnea [e]	1868	764 (75.8)	1104 (83.4)	<.001	637 (85.8)	467 (80.2)	.007
Crackles	1421	552 (54.3)	869 (64.8)	<.001	482 (64.1)	387 (65.7)	.54
Grunting	489	212 (20.9)	277 (20.7)	0.9	181 (24.2)	96 (16.4)	<.001
Nasal flaring	1515	591 (58.2)	924 (69.0)	<.001	550 (73.3)	374 (63.4)	<.001
Tachycardia [f]	1296	547 (54.0)	749 (55.9)	.35	439 (58.5)	310 (52.6)	.03
Temperature $\geq$38.5°C	539	210 (20.7)	329 (24.5)	.03	194 (25.8)	135 (22.9)	.22
Weight-for-age [g]				<.001			.004
Normal	1493	701 (69.3)	792 (59.4)		417 (55.7)	375 (64.1)	
Moderate	438	178 (17.6)	260 (19.5)		152 (20.3)	108 (18.5)	

	N	All CXRs			Abnormal CXRs		
		Normal	Abnormal	p-value [b]	Any consolidation	Other infiltrate only	p-value [b]
Severe	415	133 (13.1)	282 (21.1)		180 (24.0)	102 (17.4)	
Vaccine status [h]				0.60			.001
None	143	58 (11.2)	85 (13.1)		50 (14.5)	35 (11.6)	
Complete Hib, no PCV	225	101 (19.4)	124 (19.2)		83 (24.1)	41 (13.6)	
Complete PCV	798	360 (69.4)	438 (67.7)		212 (61.5)	226 (74.8)	
Antibiotic pretreatment [i]	953	367 (38.4)	586 (46.2)	<.001	360 (50.3)	226 (40.9)	<.001
Died within 30 days	196	66 (7.4)	130 (11.2)	.003	98 (15.3)	32 (6.2)	<.001

Abbreviations: Hib=*Haemophilus influenzae* type b conjugate vaccine; PCV=Pneumococcal conjugate vaccine; HIV=human immunodeficiency virus, WHO, World Health Organization.

[a] Restricted to cases with an interpretable chest x-ray finding (n=2358).

[b] P-value obtained from Pearson chi-square.

[c] South Africa and Zambia sites only.

[d] Hypoxemia=room air pulse-oximetry reading of <90% if at elevation (Zambia and South Africa) or <92% at all other sites. If a room air oxygen saturation reading was not available and the child was on supplemental oxygen they were also considered hypoxic.

[e] Tachypnea=respiratory rate ≥60 breaths per minute for children <2 months of age, ≥50 for children 2-11 months, and ≥40 for children 12-59 months.

[f] Tachycardia=heart rate >160 beats per minute (bpm) if age 1-11 months, >150 bpm if age 12-35 months, and >140 bpm if age 36-59 months.

[g] Weight-for-age=severe if <-3 z-scores, moderate if between -3 and -2 z-scores below WHO median.

[h] Vaccine comparison excludes those children too young (<4 months) to have completed a full biologic course of Hib and/or PCV vaccines, as these children by definition are considered to be 'unvaccinated' but are not an appropriate comparison group for those who have been vaccinated. Complete vaccination for PCV was defined as 3 or more doses, or 2 doses if there was at least 8 weeks between doses and the child was <9 months of age at enrollment or >12 months of age at the time of the first dose, or 1 or more doses if the age at any of the doses, or age at introduction, was ≥24 months. Complete vaccination for Hib conjugate vaccine was defined as 3 or more doses, or 1 or more doses for a child older than 12 months at first dose.

[i] Antibiotic pretreatment was defined by having either a positive serum bioassay or documented administration of antibiotics on the day of admission at the referral or study hospital prior to blood culture collection.

Acknowledgements:

PERCH Expert Group. William C. Blackwelder, Harry Campbell, John A. Crump, Adegoke Falade, Menno D. de Jong, Claudio Lanata, Kim Mulholland, Shamim Qazi, Cynthia G. Whitney.

Pneumonia Methods Working Group. Robert E Black, Zulfiqar A Bhutta, Harry Campbell, Thomas Cherian, Derrick W Crook, Menno D de Jong, Scott F Dowell, Stephen M Graham, Keith P Klugman, Claudio F Lanata, Shabir A Madhi, Paul Martin, James P Nataro, Franco M Piazza, Shamim A Qazi, and Heather J Zar.

PERCH Chest Radiograph Reading Panel

Readers: Dr. Kamrun Nahar, Dr. Fariha Bushra Matin, Dr. Claire Oluwalana, Dr. Bernard Ebruke, Dr. Joyce Sande, Dr. Micah Silaba Ominde, Dr. Mahamadou Diallo, Dr. Breanna Barger-Kamate, Dr. Nasreen Mahomed, Dr. David Moore, Dr. Anchalee Kruatrachue, Dr. Piyarat Suntarattiwong, Dr. Musaku Mwenechanya, Dr. Rasa Izadnegahdar, **Arbitrators:** Dr. Vera Manduku, Dr. John DeCampo, Dr. Marg DeCampo, Dr. Fergus Gleeson.

PERCH Contributors:

Bangladesh: Kamrun Nahar, Arif Uddin Sikdir, Sharifa Yeasmin, Dilruba Ahmed, Muhammad Ziaur Rahman, Muhammad Yunus, Muhammad Al Fazl Khan, Muhammad Jubayer Chisti, Abu Sadat Muhammad Sayeem, Shahriar Bin Elahi, Mustafizur Rahman; **The Gambia:** Michel Dione, Emmanuel Olutunde, Peter Githua, Ogochukwu Ofordile, Rasheed Salaudeen, David Parker; **Kenya:** Shebe Mohamed, Siti Ndaa, Micah Silaba, Neema Muturi, Angela Karani, Sammy Nyongesa, Anne Bett, Daisy Mugo, Salim Mwarumba, Robert Musyimi, Andrew Brent, James Nokes, David Mulewa, Joyce Sande, John Odhiambo, Joshua Wambua, Nuru Kibirige, Caroline Mulunda, Hellen Mjalla, Norbert Katira, Karen Dama, Loice Masha, Christine Mutunga, Mwanajuma Ngama, Stephen Mangi, Riziki Anthony, Mwarua Yubu, Elijah Wakili, Benson Katana, Shoboi Mgunya, Emmanuel Mumba, Benedict Mver, George Kuria, Felix Githinji, Norbert Kihuha, Boniface Jibendi, Tahreni Bwanaali, Agustus Kea; **Mali:** Nana Kourouma, Aliou Toure, Mahamadou Diallo, Breana Barger-Kamate, Mariam Samake, Seydou Sissoko, Abdoul Aziz Maiga, Mariam Samake, Toumani Sidibe, Mariam Sylla, Aziz Diakite, Bassirou Diarra; **South Africa:** Azwidihwi Takalani, Andrea Hugo, Susan Nzenze, Ndulela Titi, Mmabatho Selela, Malebo Motiane, Minah Nkuna,Nonhlanhla Tsholetsane, Sibonsile Moya, Debra Katisi, Tondani Netshishivhe, Lerato Mapetla, Gudani Singo, Simphiwe Gasa, Cece Mgenge, Nozipho Mthunzi, Nombulelo Monedi, Tanja Adams, Shafeeka Mangera, Jeannette Wadula, Peter Tsaagane, Jenifer L. Vaughan, Sakina Loonat, Martin Hale, Sugeshnee Pather, Mariëtte Middel, Siobhan Trenor, Palesa Morailane, Ntombi Maya, Rene Sterley, Charné Combrinck, Given Malete, Lerato Qoza, Grizelda Liebenberg, Hendrik van Jaarsveld, Zunaid Kraft, Lisa-Marie Mollentze, Lourens Combrinck, Tsholofelo Mosome; **Thailand:** Sununta Henchaichon, Dr. Tussanee Amornintapichet, Dr. Somchai Chuananont,

Toni Whistler, Juraiporn Ratanodom, Patranuch Sapchookul, Ornuma Sangwichian, Sirirat Makprasert, Manoon Hirunsalee, Possawat Jorakate, Anek Kaewpan, Duangkamol Siludjai, Apiwat Lapamnouysup, Dr. Wantana Paveenkittiporn, Waraporn Ubonphen, Dr. Peera Areerat, Ms.Yupapan Wannachaiwong, Ms Tewa Faipet, Ms Punnat Natnarakorn, Ms Ahchanan Sacharone, Mr.Winai Makmool, Ms. Kanlaya Sornwong, Ms. Promporn Sansuriwong, Ms. Ratchanida Potiya, Ms. Wasana Hongsawong, Ms.Wipa Matchaikhen, Ms. Thatsanawan Chaiyabil, Ms.Piyapai Wannarach, Ms Chamaiporn Wadeesirisak, Mr. Yuttapong Norapet, Mattana Bangkung, Mr. Barameht Piralam, Sathapana Naorat, Anchalee Jatapai, Prasong Srisaengchai, Dr. Leonard Peruski, Ms.Dawan Phaensoongnoen, Ms.Tussaaorn Klangprapan, Ms.Narawadee Dumrongdee, Ms.Atchara Srithongkham, Mr. Piyawut Noinont, Ms. Pornthip Kamlee, Ms.Siyapa Mongkornsuk; **Zambia:** Justin Mulindwa, Musaku Mwenechanya, John Mwaba, Magdalene Mwale, Julie Duncan, Kazungu Siazele, Muntanga Mapeni, Emily Hammond; **Canterbury Health Laboratory, Christchurch, New Zealand:** Trevor P. Anderson, Joanne Mitchell, Rose Watt, Shalika Jayawardena**; The Emmes Corporation, Rockville, Maryland:** Mark Wolff, Megan Sanza, Omid Neyzari**.**
